# Re-Annotation of Protein-Coding Genes in the Genome of *Saccharomyces cerevisiae* Based on Support Vector Machines

**DOI:** 10.1371/journal.pone.0064477

**Published:** 2013-07-10

**Authors:** Dan Lin, Xin Yin, Xianlong Wang, Peng Zhou, Feng-Biao Guo

**Affiliations:** Center of Bioinformatics, School of Life Science and Technology, University of Electronic Science and Technology of China, Chengdu, China; Tata Institute of Fundamental Research, India

## Abstract

The annotation of the well-studied organism, *Saccharomyces cerevisiae*, has been improving over the past decade while there are unresolved debates over the amount of biologically significant open reading frames (ORFs) in yeast genome. We revisited the total count of protein-coding genes in *S. cerevisiae* S288c genome using a theoretical approach by combining the Support Vector Machine (SVM) method with six widely used measurements of sequence statistical features. The accuracy of our method is over 99.5% in 10-fold cross-validation. Based on the annotation data in Saccharomyces Genome Database (SGD), we studied the coding capacity of all 1744 ORFs which lack experimental results and suggested that the overall number of chromosomal ORFs encoding proteins in yeast should be 6091 by removing 488 spurious ORFs. The importance of the present work lies in at least two aspects. First, cross-validation and retrospective examination showed the fidelity of our method in recognizing ORFs that likely encode proteins. Second, we have provided a web service that can be accessed at http://cobi.uestc.edu.cn/services/yeast/, which enables the prediction of protein-coding ORFs of the genus *Saccharomyces* with a high accuracy.

## Introduction

Since *Saccharomyces cerevisiae* genome was first sequenced in 1996 [1], [2], the well-studied annotation of this eukaryotic organism has gone through a frequent update [3]. Many experimental studies have been published and they provide the *S. cerevisiae* genome more and more reliable reference sets. However, not all genes could be validated by experiments because many genes express only in some special conditions. Therefore, researchers may need to seek alternative methods.

Many computational approaches have been applied to predict functional ORFs and yielded various estimations. Initial sequencing of the yeast genome revealed about 6000 genes on 16 chromosomes [1]. The predicted number of genes varies dramatically based on the compositional features of coding sequences. Blandin et al. revisited the entire *S. cerevisiae* sequence using the same criteria for all 16 chromosomes [4]. They proposed that the actual protein-coding gene set of *S. cerevisiae* amounted to at least 5600 genes. Zhang & Wang [5] predicted less than 5645 genes using YZ scores to identify coding and non-coding ORFs. Wood et al.'s [6] prediction suggested 5807 genes, and the number declined to 5570 after eliminating hypothetical ORFs. Mackiewicz et al. [7] predicted 5300–5400 genes with an asymmetry model, and suggested that many predicted numbers were overestimated. Luo et al. rejected 470 spurious ORFs using the inhomogeneity index to discriminate between coding and non-coding ORFs [5]–[8].

Large-scale comparative genomic analyses within organisms in the past decade made sparkling contributions to annotation refining and updating. Specifically, independent comparative analyses between yeast and other ascomycete species [9]–[11] recommended to remove 402, 513 and 495 ORFs, respectively, from the initial predicted ORF set according to poor cross-species conservation. Those ORFs lacking evolutionary conservation are named “orphan” ORFs, and a large portion of orphans overlap with other genes [12], [13].

While a gradual and steady increment has been seen in the annotated genes verified by experimental results of transcriptome and translated proteome, there are approximately 1700 ORFs with unknown biological functions in the latest version of Saccharomyces Genome Database (SGD) [14], [15]. A study on uncategorized ORFs with biological function proposed that the majority of uncharacterized ORFs are bona fide genes according to their sequence properties [16]. In addition, Li et al. [17] suggested that many orphan ORFs, though with poor interspecies homology in comparative genome analysis and thus previously accepted as non-functional, might possess biological functions.

We sought to re-annotate the protein-coding genes in *S. cerevisiae* S288c genome using Support Vector Machines (SVMs), combined with six natural measurements in genomic research to identify bona fide genes from currently unverified ORFs based on the annotation from SGD database. Ten-fold cross-validation performed on the training dataset guarantees the accuracy of our method, and it supports coding potentials of the newly added ORFs in the SGD snapshots. Using the model trained with the optimal measurements, we estimate the number of biologically significant ORFs to be 6091, a larger number than many previous computational predictions.

## Databases and Methods

### SGD Database

The SGD [15] provides comprehensive and integrated biological information for the *S. cerevisiae* and maintains up-to-date genome annotations of *S. cerevisiae* with continuous updates from accumulated experimental results and comparative analysis. We acquired the reference genome from SGD (http://yeastgenome.org) released on 5 Jan 2010. This release includes 6603 ORFs: 4848 of them are verified genes (of which 4835 genes located on 16 chromosomes are extracted for training), 944 are uncharacterized and 811 are dubious (of which 1744 on chromosomes are extracted as test set). Additionally, the 6624 intergenic sequences from the *S. cerevisiae* S288c genome were used as negative samples in the training data sets. SGD initialized its classification system in 2003: all ORFs are assigned into one of the three categories (verified, uncharacterized and dubious) based on their coding capacity available from the published data.

### Construction of datasets

To construct the training data sets for the SVMs, all 4835 verified ORFs located on yeast's 16 chromosomes were chosen as positive samples. Among 6624 intergenic sequences, those longer than 300 bp were chosen as negative samples, and this resulted in 3515 intergenic sequences. The 300 bp length was chosen to maintain the consistency with Sharp and Cowe's threshold [18].

The uncharacterized and dubious ORFs from SGD were used as the test dataset. Because more and more orphan ORFs are found to have transcription, translation or other functions, we would like to test our method on these questionable ORFs.

### Features to assess ORF coding capacity

Many computational models with different types of features have been successfully applied into de novo identification of protein-coding genes in genome-wide research [19]. In this study, six natural and widely used features were chosen to build the SVM model. These features are listed as follows.

1) Base compositional features

Base composition asymmetry was observed by many researchers [20], [21]. The bias of specific nucleotides to different codon position was used to construct two features: nucleotide frequencies, which were calculated on all three frames of each ORF, yielding 12 parameters, and dinucleotide frequencies, which were counted on two codon positions 1 & 2, 2 & 3 and 3 & 1 using a sliding window of 3 bp, generating 48 parameters.

2) Mono-/di-codon usage features

Codon usage, i.e. frequency of natural encoding units, is a common measurement in genomic research. Since different optimal codon usage across species suggests the bias of codon adoption in genuine coding regions, we draw the measurement of codon composition using 64 features describing frequencies of all 64 codons. Similar to the 1-level Markov chain, we also described the dicodon usage with a 4096-dimensional vector using a sliding window of 6 bp.

3) Mono-/di-amino acid usage

Amino acid usage could be regarded as the degenerated codon usage by assuming an equal adoption of synonymous codons. Treating the three stop codons as a special `amino acid', we extracted 21 parameters for mono-amino acid usage and 441 di-amino acid usage parameters using a sliding window of 3 bp.

Once we finished the calculation of nucleotide/codon/amino acid usage frequencies, all the parameters were normalized onto the interval of [0, 1]. And the scale for training set normalization was stored and applied to the test set later.

### Classification Method

Support Vector Machine (SVM) has gained much popularity with wide applications in various fields [22]. In general, Support Vector Classifiers (SVCs) learn a classification problem by constructing an optimal hyper-plane with maximized margin, and predict samples by measuring the distance from where the point stands to the hyper-plane. In this work, an open-source library, libsvm, of SVM implementation written by Lin et al. was used to classify ORFs [23]. The SVM was trained with the featured parameters of the training dataset and multiple combinations of different features were exploited. When verify the effectiveness of the method, we divided all samples into ten parts with equal size, and performed 10-fold cross validation. When applying the method in dubious ORFs, a new SVM model was trained on the entire training set and was then used to the test dataset to identify protein-coding genes. Given the large samples and high-dimensional feature vectors for our classification problem, we used a linear kernel for SVM.

## Results and Discussion

### Screening on multivariate parameters

A ten-fold cross-validation was applied to screen the combinations of different parameters for the most effective set of features in identifying the protein-encoding potential of an arbitrary ORF. Using the training set consisting of 4835 verified ORFs and 3515 intergenic sequences, the cross-validation was performed on 63 combinations that may identify the accuracy of every group of gene-identifying features. In the 10-fold cross-validation, the dataset was divided into ten subsets of samples; 9 subsets were sequentially used to train the SVM and the remaining one was used for validation; each subset was guaranteed to be validated for once and the average prediction of the 10 models was adopted for the final accuracy of training. The accuracy is measured by the following two parameters,
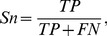
(1)which evaluates the capacity of the method in categorizing positive samples as coding genes correctly, and,
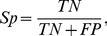
(2)which denotes the percentage in identifying non-coding sequences correctly.

In Eqs 1 and 2, *TP* and *TN* denote the number of coding/non-coding sequences that were correctly identified, while *FN* and *FP* denote the number of coding/non-coding sequences that were falsely identified.

Table 1 lists the cross-validation results on all 63 combinations. Codon and dicodon usages display notable conservation compared with the other four features. While all features demonstrate fair accuracy in identifying protein-coding genes, the combination consisting of all six features, namely nucleotide and di-nucleotide compositions, mono-/di-codon usage and mono-/di-amino acid usage, as highlighted in Table 1, gave the best sensitivity and specificity. Additionally, we respectively calculate the standard deviations of the sensitivity and specificity, and get a stable sense of the classifiers as shown in Table 2. However, the best combination misidentified 15 verified ORFs (with an average length of 296.6 nucleotides) out of 4835 genes as non-coding sequences in the cross-validation. None of these 15 falsely categorized genes is longer than 400 bp while the overall average size of the verified genes is much longer, 1546.8 bp (see Table 2).

**Table 1:** Performance for all the 63 groups of measurements in cross-validation.*

Measure ments	Sn	Stdev	Sp	Stdev	Accuracy
1	97.52%	0.39%	96.68%	0.74%	97.10%
2	99.23%	0.34%	99.21%	0.46%	99.22%
3	99.71%	0.28%	99.77%	0.16%	99.74%
4	99.67%	0.24%	99.69%	0.24%	99.68%
5	96.61%	0.69%	95.66%	1.24%	96.13%
6	98.04%	0.64%	96.48%	1.05%	97.26%
1, 2	99.21%	0.38%	99.29%	0.37%	99.25%
1, 3	99.67%	0.30%	99.80%	0.19%	99.74%
1, 4	99.67%	0.28%	99.74%	0.25%	99.71%
1, 5	99.36%	0.45%	99.55%	0.31%	99.45%
1, 6	99.30%	0.38%	99.01%	0.68%	99.15%
2, 3	99.69%	0.25%	99.74%	0.16%	99.72%
2, 4	99.71%	0.28%	99.74%	0.25%	99.73%
2, 5	99.65%	0.38%	99.66%	0.26%	99.65%
2, 6	99.44%	0.37%	99.35%	0.37%	99.39%
3, 4	99.71%	0.31%	99.74%	0.26%	99.73%
3, 5	99.71%	0.28%	99.72%	0.19%	99.71%
3, 6	99.52%	0.31%	99.40%	0.44%	99.46%
4, 5	99.69%	0.30%	99.72%	0.26%	99.70%
4, 6	99.67%	0.29%	99.55%	0.37%	99.61%
5, 6	98.68%	0.57%	98.07%	0.52%	98.37%
1, 2, 3	99.67%	0.25%	99.74%	0.16%	99.71%
1, 2, 4	99.69%	0.24%	99.72%	0.25%	99.70%
1, 2, 5	99.65%	0.38%	99.63%	0.27%	99.64%
1, 2, 6	99.50%	0.42%	99.38%	0.34%	99.44%
1, 3, 4	99.69%	0.27%	99.72%	0.26%	99.70%
1, 3, 5	99.67%	0.28%	99.80%	0.19%	99.74%
1, 3, 6	99.59%	0.33%	99.46%	0.37%	99.52%
1, 4, 5	99.67%	0.28%	99.77%	0.23%	99.72%
1, 4, 6	99.63%	0.27%	99.72%	0.27%	99.67%
1, 5, 6	99.32%	0.38%	99.12%	0.56%	99.22%
2, 3, 4	99.71%	0.28%	99.77%	0.26%	99.74%
2, 3, 5	99.69%	0.25%	99.74%	0.16%	99.72%
2, 3, 6	99.67%	0.29%	99.49%	0.23%	99.58%
2, 4, 5	99.69%	0.28%	99.72%	0.29%	99.70%
2, 4, 6	99.65%	0.16%	99.72%	0.26%	99.68%
2, 5, 6	99.50%	0.36%	99.26%	0.44%	99.38%
3, 4, 5	99.69%	0.30%	99.74%	0.26%	99.72%
3, 4, 6	99.67%	0.24%	99.72%	0.31%	99.69%
3, 5, 6	99.57%	0.32%	99.46%	0.47%	99.51%
4, 5, 6	99.67%	0.28%	99.57%	0.38%	99.62%
1, 2, 3, 4	99.71%	0.28%	99.77%	0.25%	99.74%
1, 2, 3, 5	99.67%	0.25%	99.77%	0.16%	99.72%
1, 2, 3, 6	99.67%	0.29%	99.49%	0.24%	99.58%
1, 2, 4, 5	99.69%	0.28%	99.72%	0.23%	99.70%
1, 2, 4, 6	99.65%	0.26%	99.77%	0.24%	99.71%
1, 2, 5, 6	99.52%	0.39%	99.38%	0.33%	99.45%
1, 3, 4, 5	99.71%	0.28%	99.74%	0.25%	99.73%
1, 3, 4, 6	99.69%	0.26%	99.74%	0.26%	99.72%
1, 3, 5, 6	99.57%	0.33%	99.49%	0.36%	99.53%
1, 4, 5, 6	99.63%	0.27%	99.72%	0.27%	99.67%
2, 3, 4, 5	99.71%	0.28%	99.74%	0.26%	99.73%
2, 3, 4, 6	99.67%	0.28%	99.80%	0.24%	99.74%
2, 3, 5, 6	99.67%	0.26%	99.52%	0.24%	99.59%
2, 4, 5, 6	99.67%	0.26%	99.74%	0.26%	99.71%
3, 4, 5, 6	99.69%	0.24%	99.74%	0.31%	99.72%
1, 2, 3, 4, 5	99.71%	0.24%	99.74%	0.26%	99.73%
1, 2, 3, 4, 6	99.69%	0.28%	99.77%	0.20%	99.73%
1, 2, 3, 5, 6	99.65%	0.29%	99.77%	0.24%	99.71%
1, 2, 4, 5, 6	99.65%	0.26%	99.77%	0.24%	99.71%
1, 3, 4, 5, 6	99.67%	0.24%	99.77%	0.26%	99.72%
2, 3, 4, 5, 6	99.69%	0.22%	99.80%	0.24%	99.75%
1, 2, 3, 4, 5, 6^a^	99.69%	0.28%	99.80%	0.20%	99.75%

* Six measurements are represented as following: 1/mono-nucleotide frequencies, 2/di-nucleotide frequencies, 3/mono-codon composition, 4/di-codon composition, 5/mono-amino acid usages, 6/di-amino acid usages.

a The boldface letter indicates the group with highest accuracy among the 63 combinations.

**Table 2:** List of misclassified genes in 10-fold cross-validation.*

ORF ID	Gene Name	GC%	Length(bp)
YAL064W	–	37.30887	327
YDR504C	SPG3	26.30208	384
YGL032C	AGA2	40.5303	264
YJL028W	–	47.61905	336
YJR120W	–	46.72365	351
YNL269W	BSC4	40.40404	396
YOR302W	–	41.02564	78
YBR058C-A	TSC3	34.97942	243
YFL010W-A	AUA1	42.45614	285
YGL168W	HUR1	32.43243	333
YJL077C	ICS3	40.90909	396
YKL037W	AIM26	49.85994	357
YOR031W	CRS5	42.38095	210
YPL096C-A	ERI1	44.44444	207
YPL183W-A	RTC6	43.97163	282

* All the 15 misclassified ORFs (with an average length of 296.6 nucleotides) are small ORFs, which are usually difficult to identify.

### Retrospective examination of methodology

While the cross-validation on the sample sets demonstrated high accuracy, we could not simply deduce a high fidelity of our method since the prediction result on biologically significant ORFs could not be experimentally verified, at least within a short period. However, an investigation into the historical snapshots of SGD database [14], [15] could provide collateral support for the reliability of our method as each database revision updated new genes that previously lacked experimental support.

In this retrospective analysis, 7 historical snapshots from Aug. 2004 to Jan. 2010 were retrieved from the SGD database. Next, a pairwise examination between two adjacent snapshots was performed in the following steps: 1) Construct the training/testing sample sets using the verified ORFs and intergenic sequences from the older snapshot; 2) Train SVM with the constructed training set; 3) Predict protein-coding genes within the testing set using the model generated in Step 2; 4) Pick all the verified ORFs annotated in both adjacent snapshots and find the newly verified genes in the newer snapshot; 5) Compare two sets generated in Steps 3 and 4 and calculate the coverage ratio of our predicted genes against the newly confirmed genes.

By examining the prediction coverage against the priori data, we could equivalently draw an assessment of our method. Table 3 lists the results in the pairwise comparison between historical snapshots. The congruent results that all coverage ratios are over 98% suggest a high sensitivity with our method in identifying “new” genes. In the historical development in the SGD annotation, 624 new genes were introduced in the past six years, of which 616, or 98.72%, were “discovered” by our method. Overall, 8 ORFs were missed in the pairwise comparison and all are small ORFs which are usually difficult to identify.

**Table 3:** Results of retrospective examination into historical snapshots.*

Compared Snapshots	*n*_new_veri_	*n*_predicted_	Misclassified ORFs	Gene Name	Coverage%
2004–2005	127	1784	YDR504C	SPG3	99.21%
2005–2006	94	1709	YJR120W		98.94%
2006–2007	216	1614	YAL064W YJL077C	–ICS3	98.15%
			YGL168W YJL028W	HUR1 –	
2007–2008	39	1436	–		100%
2008–2009	103	1388	YKL037W YPL183W-A	AIM26 RTC6	98.06%
2009–2010	45	1317	–		100%

* *n*_new_veri_ denotes the number of verified genes newly added in updated snapshot of SGD database; *n*_predicted_ denotes the overall predicted protein-coding ORFs based on every historical version of SGD database. Take the first snapshot as example, we predict 1784 coding ORFs based on the data of 2004, which covered 99.21% of the 127 newly added verified genes in 2005.

### Parameters optimization

**Figure 1:**
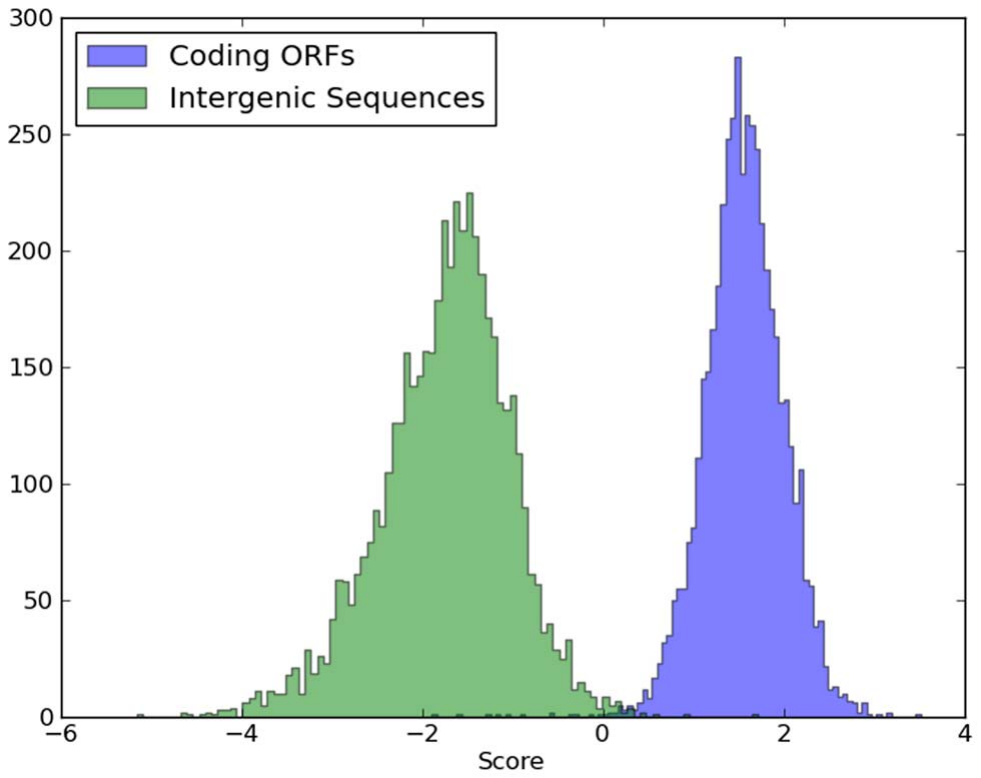
Histogram distribution of predicted scores of samples on the training set. Genes with scores <0 are predicted as non-coding genes, and >0 coding ones. As denoted in the overlapping areas, *FN* (coding genes with predicted score inferior to zero) is 15 and *FP* (intergenic sequences with score over zero) is 7. The list of 15 misclassified genes is as shown in Table 2.

By examining the raw score of samples' margin to the classifying hyper-plane, we can visualize the distribution of the predicted scores in the training set rather than the binary classification result. The distribution of the predicted scores, as given in Figure 1, demonstrates how both positive and negative sequences were classified with the trained SVM. A small overlapping region in the middle indicates those incorrectly classified samples.

We sought to optimize the classification by raising the sensitivity while not sacrificing much specificity through offsetting the threshold for decision. However, the result was not quite satisfying: specificity dropped dramatically while we shifted the threshold for a slight increment in sensitivity. This observation clearly showed that SVM, in this case, is an efficient and practical classifier that solves the classification problem well.

In addition, we optimized the combination of the penalty parameter *C* and the biased weight parameter *w*_ratio_ using a grid-searching method based on the cross-validation on the gold-standard training set. Define the *w*_ratio_ to be,
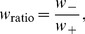
(3)where *w*___ and *w*_+_ correspond to the weight scaled on *C* for negative and positive datasets, respectively.

The optimal classification was achieved when *C* =  2^−3.5^, and *w*_ratio_  = 2^0.6^. By applying this combination of the parameters, we got the highest predicted accuracy. These values were adopted in the following prediction. Furthermore, we noticed that the specificity, compared to the sensitivity, is prone to change when parameters vary, possibly due to the dense distribution of the negative samples around the margin, which could be supported by more severe variation when the cost parameter *C* for negative samples gets smaller. As a result, it might sacrifice much of *Sp* to increase *Sn*.

### Prediction of protein-coding genes

Finally, the best-trained SVM model with the combined features of nucleotide/di-nucleotide compositions, mono-/di-codon usage and mono-/di-amino acid usage was applied on all 1744 chromosomal ORFs labeled as dubious or uncharacterized. Among 1744 ORFS, 1256 ORFs (826 uncharacterized and 430 dubious) were predicted to be protein-coding; equivalently, 488 spurious ORFs were ruled out from the yeast genome. We then estimated the overall number of protein-coding genes in *S. cerevisiae* genome to be 6091.

### Why ∼500 spurious genes were ruled out?

Using our methodology, we identified 488 spurious ORFs as non-coding (with an average length of 300.8 bp and a median length of 321 bp). Recent discovery found that many different kinds of products are generated by a pervasive transcription, which focuses mainly on small non-coding RNAs (ncRNAs) associated with promoters in eukaryotes from animals to yeast, showing that the yeast genome is almost entirely transcribed (sense and anti-sense) [24]. Among these 488 ORFs, 243 overlap with verified genes and a very high percentage (∼85%) of them are located on the antisense strands of verified genes. This observation is consistent with previous research discovery [25], where the over-annotated ORFs in the genome of the Crenarchaeon, *Aeropyrum Pernix* K1 also tend to overlap real genes on the antisense strands.

**Figure 2:**
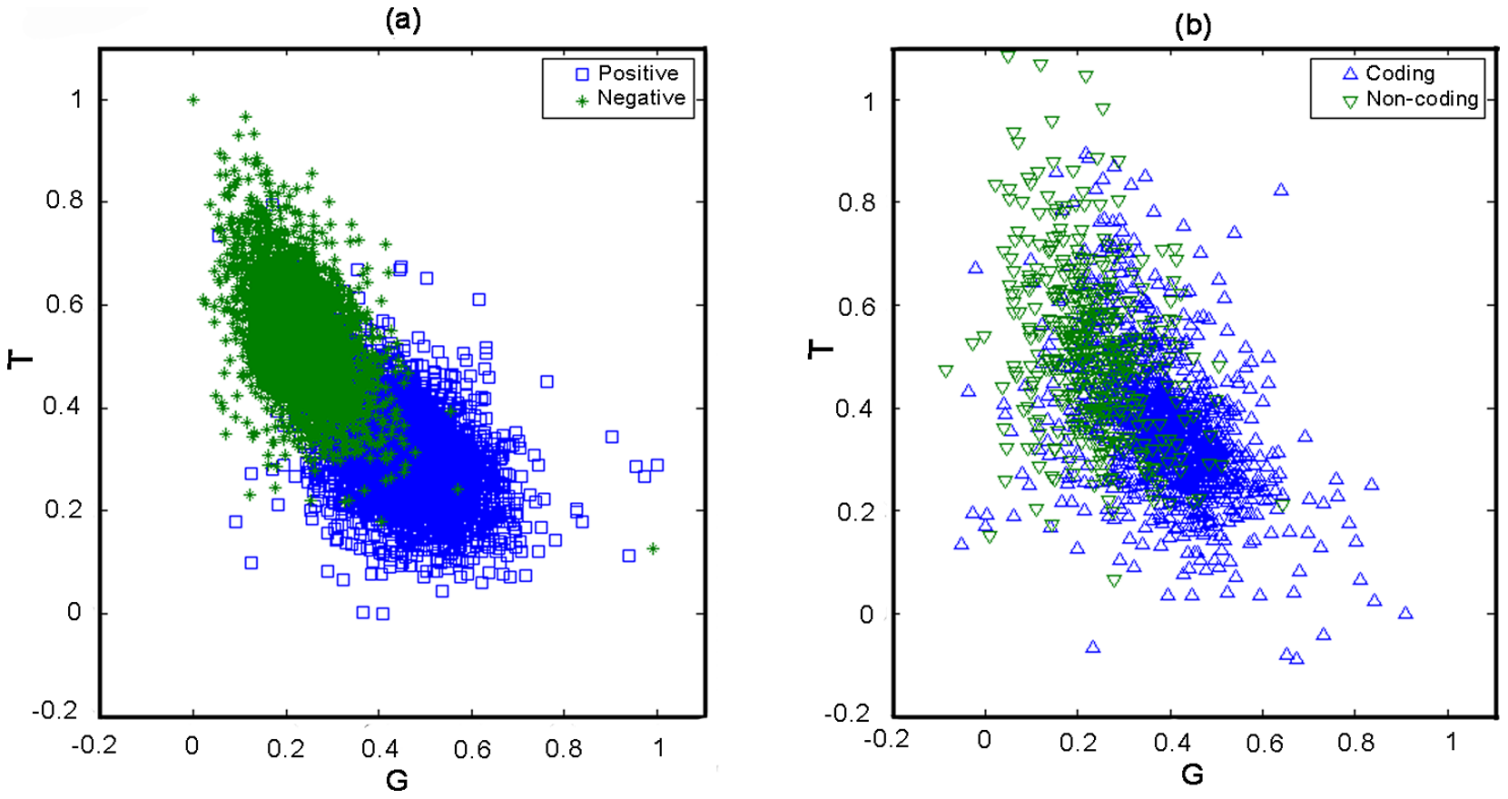
G-T nucleotide distribution on 1st codon position of all four sets of ORFs. (a) G-T distribution of 4835 positive and 3515 negative samples in training sets. (b) G-T distribution for 1256 predicted genes and 488 rejected spurious ORFs and all 1744 ORFs are those originally labeled as dubious or uncharacterized by the SGD annotation.

Looking into the features of sequence structure, we compared the distribution of nucleotide frequencies on different codon positions for all our training and test samples. As depicted in Figure 2, the G-T plane demonstrates the distribution of guanine and thymine usage on the first codon position for our datasets. Each point on the graph denotes one ORF. Figure 2(a) depicts the distinct pattern of guanine and thymine usage for positive and negative training samples, in which guanine shows predominant occupancy for genes, while thymine tends to be more frequent for non-coding ORFs, both on the first codon position. In addition, G-T distribution of our predicted coding genes is consistent with that of positive samples in training sets, and the same is true of our non-coding ORFs, which can be shown in the comparison of Figure 2(a) and Figure 2(b).

Similar results are drawn from the A-T, C-G planes where coding ORFs show predominant usage of purine bases, which is observed by other researchers as universal and conserved patterns [26]. Therefore, the congruent pattern of both verified and our predicted data supports our prediction by eliminating the 488 non-coding ORFs. Though the separating tendency appears in both figures, however, it may be noted that the separation between two types of samples in Figure 2a is much significant than that in Figure 2b. In fact, we used a total of 4682 variables to jointly discriminate the positive and negative samples. Using only two dimensions, test set are more difficult to differentiate than the training set although the classifications may have the similar precisions when using 4682 variables.

### Controversy over the number of genes

In the past decade, debates over the accurate number of protein-coding genes in *S. cerevisiae* genome are never settled. The re-annotation number of 6091 in our prediction of the protein-coding genes in *S. cerevisiae* genome is in accordance with the initial extrapolation (6200 ORFs, and 6%∼7% of which do not encode proteins) [12]. Additionally, this number is much higher than many previous estimations [4]–[6]. Compared with the previous computational genome analyses, our method demonstrates a higher accuracy in identifying ORFs with coding potential. The multivariate parameters, along with SVM as an efficient machine learning method, contribute to the high sensitivity and specificity (>99.5%), and thus may provide more reliable and accurate results for overview of the functional ORFs in *S. cerevisiae* genome.

While orphan ORFs were recommended for deletion form proteome set by comparative analyses [27], some of them are congruently abundantly transcribed compared with other genes [12]. In addition, the results from a few recent high-throughput experiments also indicated that a large portion of evolutionary non-conserved ORFs are actively transcribed or translated [17].

Comparative analyses conducted by Brachat et al. [9], Cliften et al. [10], and Kellis et al. [11] in all suggested 648 ORFs show no homology to other species as spurious. Comparison between our prediction and these comparative results shows that 295 and 248 spurious genes annotated by Cliften and Kellis, respectively, are predicted as genes with our method. After comparative analysis, these retained spurious ORFs are found to have similar nucleotide compositions with verified genes than with discarded spurious ORFs.

### Web service

The method presented in the paper has been implemented as an online web service, namely Saccharomyces SVM, which is accessible at http://cobi.uestc.edu.cn/services/yeast/. The following functions are provided: (i) An user interface shown in the first section ‘Run the Service’ to submit ORFs to predict their coding potential. (ii) An introduction about the service Saccharomyces SVM in the second section ‘Reference Implementation’, which mainly presents the methodology, the test result of our method and the instruction to use our online service. Specially, we compile a complete list of our predicted results, which contains both 1256 protein-coding genes and 488 rejected spurious ORFs with attributes like locations, lengths and annotated functions. The list is attached to http://cobi.uestc.edu.cn/resource/yeast_svm/orflist/. Using the available annotation data of *Schizosaccharomyces pombe* genome released from NCBI, we obtain an accuracy of 98% by running the service to predict its coding potential. Since *S. pombe* and *S. cerevisiae* differs in genera, we rationally recognized that the species of *Saccharomyces* will be predicted with much higher accuracy.
